# Predictors of Major Dietary Patterns Among Pregnant Women Attending Public Health Facilities in Eastern Ethiopia: A New Epidemiological Approach

**DOI:** 10.3389/fnut.2022.855149

**Published:** 2022-04-25

**Authors:** Abdu Oumer, Mihret Abraham, Aliya Nuri

**Affiliations:** Department of Public Health, College of Medicine and Health Sciences, Dire Dawa University, Dire Dawa, Ethiopia

**Keywords:** dietary pattern, food frequency, pregnant, factor analysis, ordinal logistic regression

## Abstract

**Background:**

Dietary pattern analysis is a robust statistical procedure that efficiently characterize the dietary intakes of individuals. However, there is a lack of robust dietary intake evidence beyond nutrient intake in Ethiopia. This study was to answer, what are the major dietary consumption patterns and its predictors among pregnant women in Ethiopia.

**Methods:**

A facility-based survey among 380 randomly selected pregnant women using a contextualized food frequency questionnaire (FFQ) over 1 month recall was used. The frequency of food consumption was standardized to daily frequency equivalents, and a sequential exploratory factor analysis was used to derive major dietary patterns. A multivariable ordinary logistic regression model was fitted with all its assumptions.

**Results:**

Three major dietary patterns (“fruits and animal-source foods,” “cereals, tubers, and sweet foods,” “legumes and vegetables”), explaining 65% of the total variation were identified. Women snacks (AOR = 1.93; 1.23–2.75), without food aversion (AOR = 1.59; 1.08–2.35), non-fasting (AOR = 0.75; 1.12–2.12), and receiving nutritional counseling (AOR = 1.96; 1.25–3.07) were significantly positively associated with a higher tercile of fruits and animal-source food consumption. Non-working mothers (AOR = 1.8;1.23–2.76), chronic disease (AOR = 1.88; 1.14–3.09), or received nutritional counseling (AOR = 1.33; 0.88–2.01), were fasting (AOR = 1.33;0.88–2.01), and no food cravings (AOR = 4.27;2.67–6.84), and aversion (AOR = 1.60;1.04–2.44) had significantly higher odds of consuming cereals, tubers, and sweet foods. Literacy (AOR = 1.87; 1.14–3.09), urban residence (AOR = 2.10; 1.10–3.93), low socioeconomic class (AOR = 2.68; 1.30–5.23), and skipping meals (AOR = 1.73; 1.15–2.62) were associated with higher odds of legume and vegetable consumption.

**Conclusion:**

Socioeconomic class, literacy, occupation, getting nutritional counseling, habits of food craving, food aversion, and fasting can predict a woman’s dietary pattern.

## Introduction

Pregnancy is one of the most critical nutritionally sensitive periods in human development for optimal maternal and newborn health outcomes (1). The need for macro and micronutrients in increased during pregnancy, specifically the need for protein, iron, folic acid, energy, vitamins, and other micronutrients is greatly increased. These could be addressed through increased meal frequency (at least one extra meal), diversified dietary consumption and avoiding harmful substances (2, 3). Preventing pregnancy-related over and undernutrition requires optimal nutrition from a variety of nutritious foods (4). Maternal malnutrition is strongly associated with an increased morbidity and mortality burden due to low birth weight, preterm birth, cognitive dysfunction, anemia, neural tube defects, and other negative consequences for the mother and newborns. Poor nutrition has long-term consequences, including an increased risk of chronic diseases in adulthood (4–8).

Macro and micronutrient malnutrition is a major public health concern among pregnant women in developing countries. Globally, a significantly higher number of pregnant women are victims of vitamin A deficiency (19 million) and anemia (40%) (9). Above all poor access to nutritious diet, prevailing non-communicable diseases and repeated pregnancies among women in low socioeconomic class makes malnutrition and adverse consequences more common (3). In Africa, about 23.5% of pregnant women are malnourished (10). An estimated 23.3 and 60% of women had chronic energy deficiency and zinc deficiency and its adverse consequences (11). In Ethiopia, undernutrition (38%) (12), anemia (32%), iodine deficiency (38%) (13), and vitamin A deficiency (14) were a major public health problems affecting a large number of pregnant women nationally.

Furthermore, the maternal mortality rate of 412/100,000 live births, stunting among young children (37%), and under-five mortality (43 deaths/1,000) (15) are consequences of poor maternal nutrition during embryonic life. Malnutrition is one of the key modifiable contributors to many adverse pregnancy outcomes, especially in developing countries (16, 17). On the other hand, having excessive weight gain due to extra energy consumption, increases the risk of operative delivery, obstructed labor, hypertensive disorders of pregnancy, and gestational diabetes mellitus (18, 19). Hence, the dietary practices of pregnant women are of paramount importance in maintaining optimal health (9). An unhealthy dietary pattern is also associated with gestational diabetes (20), poor fetal growth, preterm births (21), malnutrition, and other adverse consequences (22).

Due to the complexity of human dietary intakes, reporting the usual nutrient intake and/or individual food consumption is not reliable and valid approach (23) (24). A comprehensive and robust dietary intake assessment is a feasible way of characterizing the dietary habits, quality, and implications of usual intakes (25, 26). Dietary pattern analysis allow us to identify the cumulative and interactive effects of each dietary component and allows us to better characterize dietary consumption (27, 28). Individual nutrient intake assessments usually fall short of accounting for the higher multicollinearity and correlation among food groups and individual nutrients in foods, which means one cannot definitely attribute a particular nutrient or food exposure (25, 28).

**FIGURE 1:**
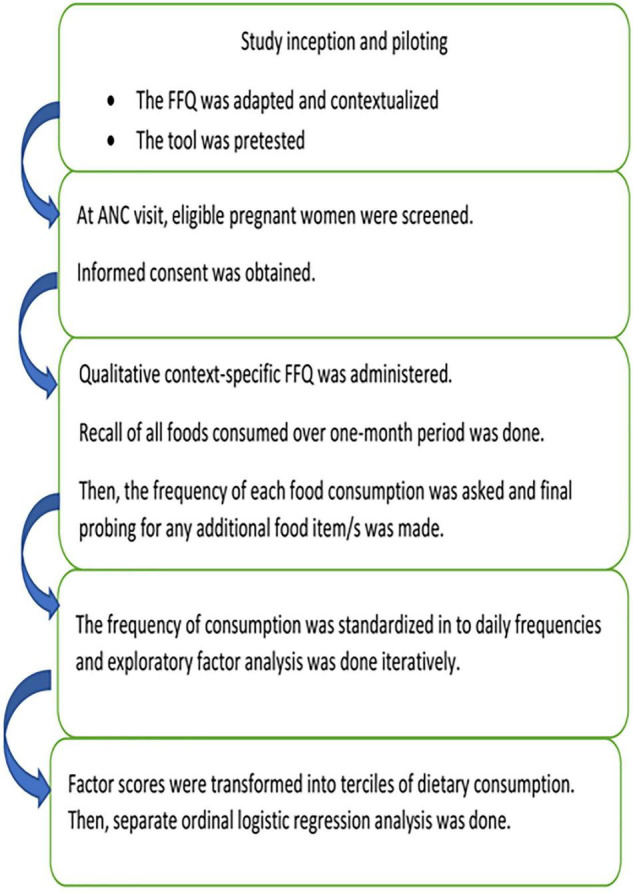
Flowchart showing overview of the study procedures, from inception and final report.

Despite the fact that concrete evidence on dietary quality and consumption patterns is crucial to designing and implementing appropriate nutrition interventions for pregnant women, such evidence is lacking in Ethiopia. Previous studies conducted (29–31) used a simple dietary practice tool which did not consider the complexity of the diet and interactions among dietary components. One study also indicated that more than half, 75% (30), and (61%) (31) of women had poor dietary practices, despite having better knowledge of optimal feeding (61%) (31). This urges further evidence on the overall dietary consumption of pregnant women and identifies the potential factors associated with poor/unhealthy dietary consumption of pregnant women. Such concrete evidence will inform a refined policy recommendation for better and healthier nutrition during pregnancy. Thus, having a reliable food frequency questionnaire (FFQ), one can capture the food consumption frequency over a period of time. This can be better treated statistically under exploratory factor analysis to identify major dietary patterns instead of individual food item consumption (26, 28). The current paper is pioneering research to characterize the dietary consumption of pregnant women in a more robust statistical approach, where such evidence is lacking, but guides further maternal nutrition interventions in the country. This study was to answer two research questions; what are the major dietary consumption patterns and predictors of these major dietary patterns among pregnant women in Ethiopia, as summarized in Figure 1.

## Materials and Methods

### Study Design and Settings

This is a health facility-based survey conducted among pregnant women visiting public health facilities in Dire Dawa, which is located 526 km (eastern parts of Ethiopia). Dire Dawa is a place for a multi-ethnic society with a diverse culture and eating habits. The total population is about 506,936, of which more than half reside in urban areas. There are diverse ethnic groups, with diverse lifestyles and dietary habits, which makes the data more representative. Agriculture in rural Dire Dawa and surrounding areas focuses on sorghum, maize, potatoes, and other cash crops. In addition, fruit and vegetable production are also present in the rural parts of the area.

### Population and Eligibility Criteria

A random sample of 380 pregnant women attending ANC in selected public health facilities were included in this study selected from all pregnant women attending public health facilities during the study period.

### Sample Size and Sampling Procedure

The sample size required to assess the dietary patterns of pregnant women was estimated using a sample size estimation formula for single proportion. We assumed a 95% confidence level, a 5% significance level, and the prevalence of poor dietary practice (66%) (32), and a 10% non-response rate. The sample size became 380. A sample size was also estimated for a cross-sectional study comparing the risks of poor dietary practice by different exposure variables, but the estimated sample was below 380. Thus, a total of 380 pregnant women having ANC visits from the selected public facilities were included.

A stratified sampling with proportional allocations (based on 3-month case load) was conducted where proportional samples were taken from urban and rural areas. Then, pregnant women visiting public health facilities at every two sampling intervals were included in this study using a systematic random sampling procedure. The first women to be interviewed were selected using simple random sampling and women visiting the health facility every two intervals were included.

### Data Collection Procedures

The data was obtained through a face-to-face interviewer administered interview technique using a pretested semi-structured questionnaire prepared in English and local languages. The tool includes sociodemographic characteristics, household asset ownership, dietary consumption, and a 40-item FFQ. The modified form of the FFQ was used to collect data on food consumption over the past 1-month recall period. The consumption frequency was captured on a nine-scale ranging from never to three times a day. The current FFQ is a relatively simple, valid, and reliable dietary assessment method that can be used to derive dietary patterns (33, 34). Evidence shows that the FFQ is a valid tool to predict micronutrient intakes (33, 35, 36). In Ethiopia, the FFQ was shown to have a high correlation with a range of micronutrient intakes and was suitable for ranking individual intakes (37), which we tried to rank based on food consumption frequencies. We utilized a 1-month recall for the FFQ, as a relatively shorter recall period allows us to capture individuals’ usual food consumption, increase response rate, and reduce recall bias and respondent burden (38, 39). A team of five trained health extension workers, supervised by two bachelor’s degree holders, collected data from Monday to Friday, when regular ANC services are provided. Data was collected on representative days, including fasting and feasting days of the week. Respondents were elicited about any foods consumed, and again, specific foods were asked about in the questionnaire one-by-one.

### Data Analysis

The cleaned data was analyzed with SPSS version 20. Categorization and recoding of variables for further analysis were done. The data was summarized into mean, standard deviation, and percentages, and presented with statistical tables and graphs. The wealth index was derived from individual household assets using principal component analysis, and factor scores were ranked to generate wealth quintiles. Items which fulfill the assumptions of PCA were considered for the analysis and determination of factor scores (40).

An exploratory factor analysis (EFA) was done with the assumption checks to identify the major dietary patterns based on frequency of consumption. First, the consumption frequency of individual foods was standardized and converted to a daily frequency based on literature. Then, to minimize the complexity of the factor analysis and to be able to identify major factors that explain the highest variation, the individual food group consumptions under pre-specified food groups were summed up in accordance with literature (41). The assumptions of EFA were checked, and those variables that did not fulfill the criteria were iteratively excluded from the analysis. The Kaiser–Meyer–Olkin (KMO) *p* > 0.05) and Bartlett’s test of sphericity (*p* < 0.05) were used to check for adequacy of the sample and the presence of significant correlation between items, to be considered for factor analysis (42). The EFA was conducted under orthogonal rotation with the varimax method, which allows one to identify interpretable, independent dietary patterns. Food groups with a factor loading above 0.5 were retained in the final EFA model. Considering the eigen value above 1, the scree plot, and/or the percentage of variation explained by the principal components, were used to decide on the final dietary patterns (43).

Furthermore, the factor score was generated using the Bartlett procedure, which is a robust and unbiased estimate of the true factor score based on the factor loading and daily consumption of food groups. The factor scores were further categorized (ranked) into three terciles (low, medium, and high) and are considered as response variables (44). The higher the factors tend to indicate more frequent or higher amounts of food consumption. Bivariable and multivariable ordinal logistic regression (OLS) models were considered to assess the factors associated with each dietary pattern tercile. The proportional odds assumption, and constant slope parameter over the three categories were checked using a test of parallel lines. Variables with a *p*-value below 0.2 in bivariable OLS were considered for multivariable OLS, where the model fitness was checked accordingly.

### Ethical Considerations

Ethical approval was obtained from Dire Dawa University’s Institutional Research Ethical Review Committee. The study was conducted in accordance with the standards and regulations of the ethical committee. A written informed consent was obtained from each pregnant woman.

## Results

### Baseline Characteristics of Pregnant Women

A total of 380 pregnant women with a median age of 28 years (IQR _25_, _75_: 24–32 years) were included in the current study. About 50.8 and 28.9% of pregnant women were Muslims and orthodox Christian in religion, respectively. Almost all (94%) were married, with only 74.4% of pregnant women being literate. The majority, 69 and 62%, of respondents were not primigravidae and from urban areas. More than half (56%) and 66% of women were not currently working and belonged to middle and lower socioeconomic groups. Regarding the dietary habits of women, 47% did not fast, 65% did no snacking, and 48% had some type of food aversion. Of the total participants, only 147 (38.7%) received nutritional counseling during their recent antenatal care visit.

### Major Dietary Patterns of Women

Before PCA, the food frequency collected under nine scales was standardized to a daily equivalent based on literature (45). Then, the foods were aggregated into major food groups and the daily frequency of consumption score was summed up (Table 1). Before performing the analysis, the PCA assumptions were double-checked. The nine-scale frequency was reclassified and scored as 0, 0.1, 0.25, 0.571, 1, and 2, depending on the consumption frequency. The sampling adequacy was checked by KMO (0.69), and there was a significant correlation among items (*X*^2^ = 546.7, *df* = 28; *P* < 0.0001). Three major dietary patterns were identified and explained 65% of the total variation in the dietary consumption of women. The identified dietary patterns had a factor loading above 0.6 in each food group (Table 1).

**TABLE 1:** Summary table for factor loading, factor scores, and variance explained by the major dietary patterns for each food groups among pregnant women in Eastern Ethiopia.

	Food groups	Factor 1^a^	Factor 2^a^	Factor 3^a^	Loading
1	Milk and meat products	0.819			0.593
2	Fruits	0.751			0.578
3	Poultry and Fish	0.768			0.584
4	Sweet foods		0.757		0.681
5	Tubers		0.737		0.644
6	Cereals		0.841		0.712
7	Pulses			0.881	0.786
8	Vegetables			0.614	0.594
Median factor score (IQR_25_, _75_)		–0.40 (–0.59, 0.24)	–0.29 (-0.88, 0.62)	–0.29 (–0.53, 0.17)	
Variation explained		26%	23%	16%	

*^a^Dietary pattern 1 refers to “animal-source foods and fruits,” dietary pattern 2 refers to “cereals, tubers, and sweety foods,” and dietary pattern 3 refers to “pulses (legumes) and vegetables”.*

Food group items with a complex structure (a higher loading for more than one factor) were excluded and we derived three major dietary patterns with dominant food item loadings for each component. These are fruits and animal-source foods, which load higher for fruits, poultry, fish, milk and meat products, cereals, tubers, and sweet foods, and pulses and vegetables. A factor score was generated using the Bartlett procedure, which is a robust and unbiased estimate of the true factor score. Then, the factor score was categorized into three terciles (low, medium, and high) (44). Three dietary patterns, including “animal-source foods and fruits,” “cereals, tubers, and sweety foods,” and “pulses (legumes) and vegetables” were identified (Table 1).

### Factors Associated With Major Dietary Patterns

The proportional odds assumption was checked for all predictor variables using the test of parallel lines, and the assumption was fulfilled for the majority of variables, except for a few variables (*p* > 0.05). After checking the assumptions, bivariable and multivariable ordinal logistic regression (OLS) were run for potential predictors of each of the identified dietary patterns. Those women who were single (COR = 1.74; 95% CI: 1.26–3.72) and living in urban areas (COR = 2.13; 95% CI: 1.44–3.14) were 1.74 and 2 times more likely to consume more fruit and animal-source foods, than those who were married and from rural areas, respectively, than those who are married and lived-in rural areas. Women’s literacy (COR = 1.86; 95% CI: 1.2–2.87) and husband’s literacy (COR = 2.1; 95% CI: 1.30–3.40) were significantly related to higher consumption of fruits and animal-source food (Supplementary Material 1).

Having nutritional counseling, fasting, snack consumption, and food aversion were significantly associated with a higher consumption of fruits and animal-source foods. Women who received nutritional counseling (AOR = 1.96; 95% CI: 1.25–3.07) and those with snacking habits (AOR = 1.93; 95% CI: 1.23–2.75) had twice the odds of consuming a higher tercile of fruits and animal-source foods as compared to their counterparts. Women who did not fast (AOR = 1.75; 95% CI: 1.12–2.12) and with no food aversion (AOR = 1.59; 95% CI: 1.08–2.35) were nearly twice as likely to consume fruits and animal-source foods (Table 2).

**TABLE 2:** Multivariable OLS for factors associated with major dietary patterns among women in Dire Dawa Ethiopia.

Factors	Categories	AOR (95% CI)	*P*-value
**Dietary pattern 1**			
Husband’s education	Literate	1.45 (0.86, 2.45)	0.169
	Illiterate	1	
Nutrition counseling	Yes	1.96 (1.25, 3.07)	0.003*
	No	1	
Fasting	Yes	1	0.008*
	No	1.75 (1.12, 2.12)	
Snack consumption	Yes	1.93 (1.23, 2.75)	0.004*
	No	1	
Food aversion	Yes	1	0.019*
	No	1.59 (1.08, 2.35)	
**Dietary pattern 2**			
Maternal occupation	Not working	1.84 (1.23, 2.76)	0.003*
	Working	1	
Chronic diseases	Yes	1.88 (1.14, 3.09)	0.013*
	No	1	
	No		
Nutrition counseling	Yes	1.54 (1.01, 2.34)	0.047*
	No	1	
Fasting	Yes	1.33 (0.88, 2.01)	0.178*
	No	1	
Food aversion	Yes	1	
	No	1.60 (1.04, 2.44)	0.032*
Food craving	Yes	1	
	No	4.27 (2.67, 6.84)	0.0001*
**Dietary pattern 3**			
Educational status	Literate	1.87 (1.14, 3.09)	0.014*
	Illiterate	1	
Maternal occupation	Not working	1	0.143
	Working	1.38 (0.9, 2.12)	
Residence	Urban	2.10 (1.10, 3.93)	0.027*
	Rural	1	
Wealth status	Poor	2.68 (1.30, 5.23)	0.008*
	Middle	2.68 (1.32 5.46)	0.007
	Wealthy	1	
Skip meals	Yes	1.73 (1.15, 2.62)	0.009
	No	1	

*Statistical significance declared at p-value below 0.05. 1 refers to the reference category, where the estimated odds ratio is compared to. AOR, Adjusted odds Ratio. Dietary pattern 1 refers to “animal-source foods and fruits,” dietary pattern 2 refers to “cereals, tubers, and sweety foods,” and dietary pattern 3 refers to “pulses (legumes) and vegetables”.

Women who were not currently working (AOR = 1.84; 95% CI: 1.23–2.76), who received nutritional counseling (AOR = 1.54; 95% CI: 1.01, 2.34), with no food aversion (AOR = 1.60; 95% CI: 1.04–2.44) and craving (AOR = 4.27; 95% CI: 2.67–6.84), and who had chronic disease (AOR = 1.88 (1.14–3.09) had 1.8–1.54, 1.6, 4.3, and 1.9-fold higher odds of consuming cereals, tubers, and sweet foods, respectively. In addition, maternal educational status, wealth status, residence, and meal skipping were significant predictors of higher consumption of food from legumes and vegetables (Table 2).

## Discussion

In this current paper, we identified three major dietary patterns and predictors of each dietary pattern among pregnant women in east Ethiopia. These are “animal-source foods and fruits,” “cereals, tubers, and sweety foods,” and “pulses (legumes) and vegetables.” Studies have shown that women’s dietary patterns are linked to the risk of adverse pregnancy outcomes like low birth weight, preterm birth, and maternal obesity and diabetes risks (23, 46, 47). Also, increased intake of Mediterranean and dietary approach to stop hypertension (DASH) diets, rich in fruits, cereals, and vegetables showed to lower the risk of hypertension and gestational diabetes mellitus (GDM) (46). In this study, we found that cereal consumption was significant, while fruit and vegetable consumption was quite low. This can potentially predispose to prevailing micronutrient deficiencies (vitamin A, iron, folic acid, and Zinc deficiency). This is evidenced by the higher prevalence of malnutrition (24%) (10) and iron deficiency anemia (32%) (48) among pregnant women in Ethiopia.

Another study from northern Ethiopia showed that 38.4 and 61% of pregnant women had poor knowledge and poor dietary practices (49). Only less than one-fifth of pregnant women (19.6%) had good dietary practices (50). This widespread poor knowledge of proper nutrition, coupled with a higher illiteracy rate and poor socioeconomic status, predispose pregnant women to poor dietary habits and prevailing malnutrition. On the other hand, a dietary pattern with non-nutritious and sweet food consumption predisposes women to obesity, impaired blood glucose, GDM, and other non-communicable diseases, especially in high socioeconomic class women (23, 46, 51).

Some baseline characteristics, such as poor socioeconomic status (AOR = 2.68) and being from urban areas (AOR = 2.10) were positively associated with a higher consumption of legumes and vegetables. For people living in low-income households, staple cereals contribute significantly to daily energy and nutrient intake and are relatively inexpensive food items (52). However, access to animal-source foods which showed over 62% price inflation, and fruits is non-affordable for the majority of the poor in Ethiopia, which significantly hinder access for consumption (53, 54). Thus, pregnant women tend to have less diverse and monotonous diets, which don’t allow them to fulfill increased physiological requirements of pregnancy for better pregnancy outcomes (54, 55).

Dietary behaviors of pregnant women in relation to food aversion and snacking habits are associated with the major dietary patterns. This study found that women with a snacking habit (AOR = 1.93; 95% CI: 1.23–2.75), but no food aversion (AOR = 1.59; 95% CI: 1.08–2.35) had higher consumption of fruits and animal-source foods. It is known that pregnant women’s energy and nutrient requirements are high, where they need an extra meal per day. Thus, women with snacks tend to opt for fruits and animal-source foods, which are rich sources of bioavailable minerals and vitamins for optimal neonatal growth (51). On the other hand, extra energy consumption from animal source foods might be related to increased obesity and its complications, as it is related to higher trans-fat consumption from fried foods and excessive weight gain during pregnancy (56). Another study showed that food aversion was common (69%), where cereals were the most commonly averted food groups (57). In this study, 52% of women had a habit of food aversion, suggesting that such harmful cultural practices usually predispose women to malnutrition, low birth weights, and other adverse maternal and newborn health outcomes (58).

Furthermore, women who do not have a habit of food craving (AOR = 4.27; 95% CI: 2.67–6.84) or aversion (AOR = 1.60) had a higher likelihood of eating cereal, tubers, and sweet foods. It is clear that habits of pica, food aversions, and cravings predispose women to low food intakes and ultimately malnutrition in developing countries. Thus, pregnant women to avoid nutritious foods such as animal-source foods, cereals, and legumes, aggravating the risk of micronutrient deficiencies (8, 57). Hormonal changes during pregnancy and culturally specific food taboos are the main drivers of consumption of non-nutritious foods and non-food items (59). Furthermore, such dietary restrictions have been linked to anemia, preeclampsia (60), low birth weight, and delayed child development (61). It is also associated with increased risk of chronic non-communicable diseases (hypertension, diabetes, cancer, and strokes) as well (62). It is mainly due to the fact that mothers usually crave high energy, fatty, and sweet foods, which are unhealthy foods (63). This will also adversely decrease zinc, iron, and other mineral bioavailability and increase occurrence of anemia and zinc deficiency (60%) (11).

In this study, women who received nutritional counseling were more likely to have a higher fruits and animal source food consumption. Also, more than half (61%) of women did not receive dietary counseling during the recent ANC visit. This indicates the need for integrated dietary counseling, which can help to pregnancy related physiological food craving, aversion, and promoting a healthy dietary consumption for better life (64). Studies also indicated that it is linked to a reduced risk of obesity and undernutrition among women (65–67). In addition, evidence from Ethiopia showed that women who received dietary counseling had 7–8 times better dietary practices (AOR = 7.2; 95% CI: 4.49, 11.49). These urges for enhanced comprehensive and targeted dietary counseling for pregnant women should be strengthened for a better maternal and fetal nutrition in Ethiopia (8).

Finally, it would be good to consider FFQ with the inclusion of comprehensive food items would be helpful to characterize and study the link with different health outcomes at a population level. Furthermore, using a quantitative FFQ would help to quantify the amount of nutrients consumed per day and it may allow to triangulated with other dietary assessment approaches. The current study involves an ethnically diverse population with diverse cultures and food consumption behaviors, which can potentially supplement targeted nutrition interventions in the area. Beyond this, such types of robust dietary assessments approaches targeting women would have a great policy implications than individual food and nutrient intake assessments, which are victims of multicollinearity and systematic errors (24, 26, 27).

### Limitations of the Study

The finding of this study should be thought in the light some inherent limitations of the paper. despite strong measures during data collection to control for social desirability, the tendency of the respondents to overreport their dietary consumptions could not be ruled out. In addition, the result of this study strongly stands on the fulfillments of the assumption of PCA, which may not hold true for all assumptions. Due to the multicultural and diverse nature of the community, minor food items n consumed by the majority might not be captured in the FFQ used for this study.

## Conclusion

Generally, three major dietary patterns, composed of cereals and tubers, legumes and vegetables, and fruits, explain the major variation in dietary consumption of pregnant women were identified. Consumption of fruits, animal-source foods, and vegetables is by far too low. Socioeconomic classes, fasting, having dietary counseling, habits of food craving aversion, and meal skipping were important predictors of the dietary patterns of pregnant women. Dietary pattern analysis can be easily used to characterize the diet of such diverse population in Ethiopia and allows to associate with many functional outcomes.

## Recommendations

We strongly recommend enhanced targeted and guided dietary counseling for pregnant women during the regular ANC visits. Also, health professionals’ capacity to effectively counsel women should be strengthened through regular capacity-building schemes. On the other hand, measures to enhance the economic capacity of individuals and mechanisms to increase the affordability of nutritionally-rich foods through wide-scale macro and microeconomic interventions. Dietary counseling messages should be designed in a way to address culture-specific unhealthy dietary behaviors that hinder nutrition-dense food consumption. Finally, we recommend further dietary assessments to consider dietary pattern analysis in the characterization of dietary consumption in addition to the usual food and nutrient intake analysis.

## Data Availability Statement

The original contributions presented in the study are included in the article/Supplementary Material, further inquiries can be directed to the corresponding author/s.

## Ethics Statement

The studies involving human participants were reviewed and approved by the DDU IRB. The patients/participants provided their written informed consent to participate in this study.

## Author Contributions

MA participated in conceptualization, fund acquisition, design, data curation, resource, project administration, data analysis, writing a report, and reviewing and approving the manuscript. AO participated in conceptualization, design, validation, supervision, methodology, data acquisition, data preparation, data visualization, and formal data analysis and contributed to writing the draft manuscript, manuscript preparation, reviewing, and submitting the manuscript. AN participated in drafting the manuscript, critically reviewing, and editing the drafted manuscript. All authors reviewed and approved the final manuscript and decided on the journal to submit to.

## Conflict of Interest

The authors declare that the research was conducted in the absence of any commercial or financial relationships that could be construed as a potential conflict of interest.

## Publisher’s Note

All claims expressed in this article are solely those of the authors and do not necessarily represent those of their affiliated organizations, or those of the publisher, the editors and the reviewers. Any product that may be evaluated in this article, or claim that may be made by its manufacturer, is not guaranteed or endorsed by the publisher.

## Abbreviations

ANC: Antenatal Care
A/COR: Crude odds ratio
CI: Confidence Interval
DASH: Dietary Approach to Stop Hypertension
EFA: Exploratory Factor Analysis
FFQ: Food Frequency Questionnaire
GDM: Gestational Diabetes Mellitus
KMO: Kaiser-Meyer-Olkin
OLS: Ordinal logistic regression
PCA: Principal Component Analysis.
