# Supplementary material for: Predictors of Major Dietary Patterns Among Pregnant Women Attending Public Health Facilities in Eastern Ethiopia: A New Epidemiological Approach
Source: Front Nutr. 2022 Apr 25;9:855149. doi: 10.3389/fnut.2022.855149 (PMC9085216; doi:10.3389/fnut.2022.855149)
Supplement: Supplementary file 1 [file Table_1.DOCX]

Supplementary table 1; factors associated with identified dietary patterns of pregnant women in east Ethiopia under the bivariable ordinal logistic regression model.

| Factors | Categories | COR (95% CI) | | |
| --- | --- | --- | --- | --- |
|  |  | dietary pattern_1 | dietary pattern_2 | dietary pattern_3 |
| Age group | 15-24 years | 0.92(0.53, 1.59) | 0.78(0.44,1.36) | 0.54(0.31, 0.94) * |
|  | 25-34 years | 1.06(0.65, 1.72) | 0.42(0.26, 0.70) * | 0.62(0.38, 1.02) |
|  | >=35 years | 1 | 1 | 1 |
| Marital status | Single | 1.74(1.26, 3.72) * | 1.76(0.81, 3.80) | 1.97(0.90, 4.28) |
|  | Married | 1 | 1 | 1 |
| Educational status | Literate | 1.86(1.2, 2.87) * | 0.49(0.32, 0.75) * | 2.78(1.80, 4.33) * |
|  | illiterate | 1 | 1 | 1 |
| Maternal occupation | not working | 1.10(0.75, 4.84) | 2.21(1.51, 3.23) * | 1 |
|  | working | 1 | 1 | 1.60(1.10, 2.32) * |
| Husband’s education | Literate | 2.1(1.30, 3.40) * | 0.49(0.3, 0.79) | 3.48(2.11, 5.75) * |
|  | Illiterate | 1 | 1 | 1 |
| Residence | Urban | 2.13(1.44, 3.14) * | 1.4(0.96, 2.10) | 4.15(2.76, 6.17) * |
|  | Rural | 1 | 1 | 1 |
| Wealth status | Poor | 1.24(0.79, 1.94) | 2.06(1.31, 3.24) * | 3.90(2.44, 6.27) * |
|  | Middle | 2.52(1.58, 4.03) * | 1.61(1.01,2.53) * | 4.38(2.70, 7.11) * |
|  | Wealthy | 1 | 1 | 1 |
| Primigravid | Yes | 1.46(0.97, 2.20) | 1.97(1.31, 2.96) * | 0.81(0.54, 1.21) |
|  | No | 1 | 1 | 1 |
| Family size | <5 | 1.21(0.83, 1.78) * | 1.80(1.23, 2.69) * | 1.12(0.76, 1.64) |
|  | >=5 | 1 | 1 | 1 |
| Chronic diseases | Yes | 1 | 1.49(0.93, 2.38) | 1 |
|  | No | 1.01(.62, 1.61) | 1 | 1.30(0.82, 2.09) |
| Khat chewing | Yes | 1 | 1 | 1 |
|  | No | 1.21(0.82, 1.78) | 0.53(0.37, 0.88) * | 0.74(0.50, 1.09) |
| ANC before | Yes | 1.23(0.87, 1.86) | 0.57(0.39, 0.85) * | 1.18(0.81, 1.63) |
|  | No | 1 | 1 | 1 |
| Nutrition counseling | Yes | 3.28(2.20, 4.80) * | 1.54(1.05, 2.25) * | 1.70(1.15, 2.5) * |
|  | No | 1 | 1 | 1 |
| Fasting | Yes | 1 | 1.06(0.73, 1.54) * | 1 |
|  | No | 2.63(1.79,3.84) * | 1 | 2.04(1.4, 2.97) * |
| Snack consumption | Yes | 2.92(1.95, 4.38) * | 1.49(1.01, 2.19) | 1.10(0.75, 1.61) |
|  | No | 1 | 1 | 1 |
| Skip meals | Yes | 1 | 0.37(0.25, 0.54) | 1.35(0.93, 1.96) |
|  | No | 1.64(1.13, 2.39) * | 1 | 1 |
| Food aversion | Yes | 1 | 1 | 1 |
|  | No | 1.60(1.10, 2.32) * | 2.82(1.93, 4.14) * | 1.10(0.75, 1.57) * |
| Food craving | Yes | 1 | 1 | 1 |
|  | No | 1.38(0.93, 2.05) * | 5.52(3.57, 8.53) * | 1.06(0.72, 1.58) |

* Statistical significance declared at p-value below 0.05. 1 refers to the reference category, where the estimated odds ratio is compared to. *COR-refers to Crude Odds Ratio; dietary pattern 1 refers to "animal-source foods and fruits,"; dietary pattern 2 refers to "cereals, tubers, and sweety foods," and dietary pattern 3 refers to "pulses (legumes) and vegetables."*
